# Lactose Intolerance and Symptom Pattern of Lactose Intolerance among Healthy Volunteers

**DOI:** 10.5005/jp-journals-10018-1156

**Published:** 2016-07-09

**Authors:** Madhusudan Saha, Irin Parveen, Bimal Chandra Shil, Shasanka Kumar Saha, Ranjit Kumar Banik, Monojit Majumder, Mahjuba Umme Salam, ASM Nazmul Islam

**Affiliations:** 1 Department of Gastroenterology, North East Medical College, Sylhet, Bangladesh; 2 Department of Gastroenterology, Enam Medical College, Savar, Dhaka, Bangladesh; 3 Department of Gastroenterology, Sir Salimullah Medical College and Mitford Hospital, Dhaka, Bangladesh; 4 Department of Biochemistry, MAG Osmani Medical College, Sylhet, Bangladesh; 5 Department of Medicine, Sylhet Women’s Medical College, Sylhet, Bangladesh; 6 Department of Gastroenterology, Sir Salimullah Medical College and Mitford Hospital, Dhaka, Bangladesh

**Keywords:** Borborygmi, Healthy volunteers, Lactose intolerance.

## Abstract

**Aims:**

To see the prevalence of lactose intolerance (LI) and related symptoms following oral lactose challenge in healthy volunteers.

**Materials and methods:**

Symptoms of abdominal pain, nausea, borborygmi, flatulence, and diarrhea were noted for 24 hours and blood glucose was estimated at 0 hour and 30 minutes after 25 gm oral lactose load to healthy volunteers. Failure to rise blood glucose level ≥ 1.1 mmol/l at 30 minutes after lactose intake from fasting level was taken as lactose malabsorption (LM), i.e., LI.

**Results:**

A total of 166 volunteers (123 males, 43 females) with a mean age 34.78 ± 11.45 years participated in this study. Lactose intolerance was found among 85.54% (n = 142, M = 104, F = 38). The main symptoms of LI were diarrhea (n = 83, 58.4.0%), borborygmi (n = 81, 57.04%), abdominal pain (n = 35, 24.65%), and flatulence (n = 27, 19.0%).

**Conclusion:**

Lactose intolerance among healthy adults may be common in Bangladesh. Diarrhea and borborygmi were mostly associated symptoms of LI.

**How to cite this article:**

Saha M, Parveen I, Shil BC, Saha SK, Banik RK, Majumder M, Salam MU, Nazmul Islam ASM. Lactose Intolerance and Symptom Pattern of Lactose Intolerance among Healthy Volunteers. Euroasian J Hepato-Gastroenterol 2016;6(1):5-7.

## INTRODUCTION

Lactose malabsorption (LM) or lactose intolerance (LI) is a “physiologic problem and is attributable to an imbalance between the amount of ingested lactose and the capacity for lactase to hydrolyze the disaccharide”.^1^ Lactose intolerance in adults is common among Africans,^2^ Jews,^3^ Asians,^4^ and other Orientals.^5^ In subjects with LM, undigested lactose is fermented by colonic flora causing diarrhea, abdominal pain, and flatulence.^6^ The severity of symptoms depends on the degree of lactase deficiency,^7^ the amount of lactose ingestion,^8^ age,^9^ ethnicity,^10^ and the gastrointestinal transit time.^7^ In subjects with LI most people due to colonic adaptation to regular lactose ingestion can ingest up to 6 to 12 gm lactose (120–240 mL milk) without developing symptoms.^11,12^

We have paucity of data on LI. A study by Alam et al in Dhaka reported 67.5% prevalence of LI in patients with irritable bowel syndrome (IBS) diagnosed by Rome II criteria.^11^ In another study, LM among Bangladeshi village children was about 80% over 36 months of age, but none of the children under 6 months of age had LI.^12^

No gold standard is available for the diagnosis of LM. Lactose hydrogen breath test (H2-BT) is considered the most accurate noninvasive test to diagnose LI. Lactose tolerance test (LTT) has a reasonable sensitivity and specificity and there is good agreement between H2-BT and LTT.^13^ But data regarding the prevalence and symptom pattern of LI among healthy adults in Bangladesh is inadequate. The present study was designed to find out the prevalence of LI and symptoms after ingestion of 25 gm lactose among the apparently healthy volunteers in the northeastern part of Bangladesh.

## MATERIALS AND METHODS

Subjects in the vicinity of North East Medical College, Sylhet, were addressed through local leaders to volunteer to participate in the present study. A total of 174 apparently healthy volunteers were enrolled in this study. Not more than two members of same family were enrolled. Informed consents were taken from the participants. Persons having fasting blood glucose level above 7.0 mmol/l, IBS (by symptom criteria), or other organic GI disorders were excluded from this study. The study was approved by the ethical committee of North East Medical College and carried out from June 2014 to October 2014. Lactose tolerance test was performed on each of these subjects using 25 gm lactose in 500 mL water after overnight fasting. Two venous samples of blood, fasting and 30 minutes after lactose intake, were taken from each subject and blood glucose was estimated. The failure of blood glucose to rise by > 1.1 mmol/l from the fasting value at 30 minutes after lactose ingestion was considered an abnormal LTT result.^14^ The symptoms developed after lactose intake were recorded from the participants for 24 hours by telephone call.

Statistical analysis was done using Statistical Package for the Social Sciences (SPSS) 16 version with significance level set at ≤ 0.05. The chi-squared test was utilized to analyze differences between proportions. Differences in the mean age of patients with positive and negative breath test were compared by using the unpaired Student’s t-test.

## RESULTS

A total of 174 apparently healthy volunteers took part in this study. Among them 8 were excluded due to high level of fasting blood glucose (> 7 mmol/l). Among the remaining 166 participants, 123 (74.1%) were male and 43 (25.9%) were female. The age of volunteers varied from 23 to 80 years with a mean age of 34.78 ± 11.45 years (Table 1).

**Table 1:** **Table 1:** Demographic features and symptom prevalence among lactose malabsorbers and lactose non-malabsorbers

		*lactose malabsorbers*		*lactose non-malabsorbers*		*Total/%*		*p-value*	
*Volunteers*		123 (73.1%)		43 (25.9%)		166 (100)			
Male		104 (84.6%)		19 (15.45%)		123 (73.1)		0.623	
Female		38 (88.4%)		5 (11.63%)		43 (25.9)			
*Mean age (18–80 years)*		35.19 ± 11.656		32.50 ± 9.004		34.84 ± 11.358		0.301	
*BMI*		20.88 ± 3.97		21.28 ± 5.91		21.64 ± 3.51		0.694	
*Symptom pattern*									
Diarrhea		83 (58.45%)		9 (37.5%)		92 (55.42)		0.170	
Borborygmi		81 (57.04%)		10 (41.66%)		91 (54.82)		0.336	
Flatulence		27 (19.0%)		5 (20.83%)		32 (19.27)		0. 771	
Abdominal pain		35 (24.65%)		7 (29.16%)		42 (25.3)		0.440	
Nausea		5 (3.5%)		1 (4.16%)		6 (3.6)		0.580	
*Number of symptoms*									
None		26 (18.30%)		7 (29.16%)		33 (19.87)		0.562	
1 symptom		38 (26.76%)		5 (20.83%)		43 (25.9)			
2 symptoms		51 (35.91%)		5 (20.83%)		56 (33.7)			
3 symptoms		24 (16.9%)		4 (16.66%)		28 (16.9)			
4 symptoms		2 (1.4%)		0		2 (1.2)			
≥ 6 symptoms		3 (2.1%)		1 (4.16%)		2 (1.2)			

A total of 142 (85.54%) subjects were found to be affected by LM. The mean blood glucose rise of the LI and non-LI subjects were 0.39 ± 0.402 and 1.600 ± 0.516 respectively, p = 0.0001). Lactose intolerance was found to be equally prevalent in both sexes (male = 104, 84.6%; female = 38, 88.4%, p = 0.623) (Table 1). The mean age and BMI of subjects with LI and non-LI were similar (Table 1).

The most common symptoms experienced by the participants having LI were diarrhea (n = 83, 58.45%), followed by borborygmi (n = 81, 57.04%) (Table 1). The sensitivity, specificity, and predictive values of individual symptoms are presented in Table 2. Diarrhea has the highest sensitivity (58.45%) and a positive predictive value of 90.22%. Next to it was borborygmi. Regression analysis showed that among the symptoms associated with LI, flatulence was the most common (OR 1.532). Values are presented in Table 3.

**Table 2:** **Table 2:** Sensitivity, specificity, PPV, and NPV of major symptoms after lactose intake

*Symptoms*		*Sensitivity*		*Specificity*		*PPV*		*NPV*		*PLR*		*NLR*	
Diarrhea		58.45%		62.5%		90.22%		20.27%		1.56		0.66	
Borborygmi		57.04%		58.33%		89.01%		18.66%		1.37		0.74	
Flatulence		19.01%		79.16%		84.38%		14.18%		0.91		1.03	
Abdominal pain		24.3%		70.83%		83.3%		13.70%		0.83		1.07	
Nausea		3.5%		95.83%		83.33%		14.38%		0.84		1.00	

PPV: Positive predictive value; NPV: Negative predictive value; PLR: Positive likelihood ratio; NLR: Negative likelihood ratio

**Table 3:** **Table 3:** Association of major symptoms with lactose malabsorbers

								*95.0% CI for OR*			
*Symptom*		*Crude OR B*		*Significance p*		*OR Exp(B)*		*Lower*		*Upper*	
Abdominal pain		0.225		0.670		1.253		0.444		3.535	
Borborygmi		–0.191		0.713		0.826		0.299		2.282	
Flatulence		0.427		0.497		1.532		0.448		5.245	
Diarrhea		–0.678		0.196		0.507		0.181		1.419	
Nausea		–0.029		0.982		0.971		0.082		11.468	

A total of 33 (19.87%) persons did not develop any symptoms during the monitoring period and among these 7 (21.21%) had a negative LTT. Table 1 shows that prevalence of LI was found to increase from subjects developing no symptom (78.8%) to subjects developing up to four symptoms (100.0) following lactose load. Subjects developing two (8.9% *vs* 91.1%, p = 0.492) and three (14.4 *vs* 85.7%) symptoms following lactose load mostly belonged to the LI group.

## DISCUSSION

Lactose intolerance is a genetically programmed decrease of lactase level in adult. Approximately 70% of the world population has primary lactase deficiency.^15^ Symptoms of lactose maldigestion are diarrhea, abdominal pain, and flatulence.^6^ The severity of symptoms depends on the degree of lactase deficiency,^7^ the amount of lactose ingestion,^8^ age,^9^ ethnicity,^10^ and the gastrointestinal transit time.^7^ Of these ethnic and genetic variations are of major importance.

According to this study, LI among apparently healthy adult volunteers was 85.54% (male 104, 84.6%; female 38, 88.4%). Lactose malabsorption is common among Asians,^4^ South Asians,^16^ and Africans.^2^ In some Asian countries it is almost 100%. Reports from the southern part of India show that its prevalence is between 60 and 70%, but it is lower (20–30%) in the northern part of India.^17^ The degree of Indo-Aryan migration and intermixing with the native population is the possible cause of this dissimilarity in prevalence. From this study, prevalence of LM in our country is higher than that in Indians, which may be explained similarly. But this result is similar to that of Malays (88%) and Chinese (91%) and Indians (83%) residing in Malaysia.^18^

There are several limitations of this study. Due to a lack of facility, the most accurate noninvasive test H2-BT could not be done to diagnose LM. Genetic testing of subjects with LM was not feasible in our setting. A good number of subjects developing symptoms escaped diagnosis with LTT. It is assumed that the yield could be higher if several methods could be employed simultaneously. Despite limitations the result of this study would definitely help our clinicians for better management of their patients. The findings of this baseline study will help future studies with appropriate investigations, larger sample size, and new dimensions.
